# Coworking Spaces: The Better Home Office? A Psychosocial and Health-Related Perspective on an Emerging Work Environment

**DOI:** 10.3390/ijerph16132379

**Published:** 2019-07-04

**Authors:** Swantje Robelski, Helena Keller, Volker Harth, Stefanie Mache

**Affiliations:** 1 Institute for Occupational Medicine and Maritime Medicine (ZfAM), University Medical Center Hamburg-Eppendorf (UKE), Seewartenstraße 10, Haus 1, 20459 Hamburg, Germany; 2 Department of Health, City of Kiel, Fleethörn 18-24, 24103 Kiel, Germany

**Keywords:** coworking space, home office, new ways of work, strain, stress

## Abstract

With the ongoing flexibilization of work, new trends concerning work outside the company’s premises such as coworking spaces are on the rise. Coworking spaces are designed to offer collaboration and community in furnished and equipped workspaces on a rental base. There is a growing body of scientific literature on coworking spaces with empirical results of qualitative and quantitative research. The present study adds to the latter by examining psychosocial demands experienced by coworkers in Germany based on a quantitative survey (*n* = 112). Among coworkers the home office was or still is another frequently used workplace. However, can the coworking space be seen as a better alternative to the home office in terms of work- and performance-related, social, environmental and health-related aspects? Results showed moderate to low psychosocial demands regarding quantitative workloads. Compared to the home office, the coworking space proved to be the preferred work arrangement. Results are discussed with regard to current literature and workplace design. In conclusion, coworking spaces can be seen as an alternative to the home office that was highly valued in the present sample. It is recommended to further emphasize aspects of work environment and ergonomics in order to create health-promoting and satisfying workplaces.

## 1. Introduction

Technological developments enable an increasing digitalization as well as a growing globalizing endeavor. This has always been accompanied by the reorganization of work. Nowadays, these developments lead to significant changes in flexibility, so that work outside the organization’s office becomes even more present in terms of temporal and spatial flexibility. Proposed benefits include an improved work–life balance or an increased sense of autonomy. At the same time, potential risks such as increasing demands, time pressure and work intensity must be taken into account [1].

Telework has been used as hypernym for different kinds of work arrangements outside the conventional office and can thus be seen as an “early form of virtual work” [2], (p. 384). As has been pointed out by Messenger and Gschwind [3], the concept of “telework has evolved constantly over four decades from the crude initial desire to reduce commuting costs to the mobilization of office work and finally to the virtualization of a whole new mode of work.” (p. 200). The virtualization of work has also been described by Morganson et al. [4] as telework arrangements. Although first developments regarding telework arrangements have been noticed already in the 1970s and 1980s, it has gained even more importance in the last decades [5,6].

Early forms of telework have been characterized by stationary information and communication technology (ICT) equipment that has been often used from home. An example of which is the home office where ICT is provided by the employer so that employees are able to work from home with varying frequency and duration. Recent developments in telework exploit all means of modern ICT, thus enabling even more flexible work arrangements—working anytime anywhere. With the increasing workforce of virtual workers, new concepts for office spaces and cooperation emerge: Coworking spaces. 

While there is an immense body of literature on health-related aspects of teleworking in a home office, little is known about possible health-related outcomes of working in coworking spaces. 

As Keller et al. [7] point out, coworking spaces offer some promising characteristics that might be advantageous in comparison to working in a home office, e.g., concerning social isolation. At the same time, potential risk factors include noise and privacy conflicts. 

The Global Coworking Survey (GCS)—currently the biggest survey among operators and members of coworking spaces conducted by Deskmag—suggests that about 45% of the coworking space-users have been working in home offices previously. Additionally, about 82% of coworkers work at least partly outside the coworking space and predominantly at home (67%) [8]. The aim of this study was therefore to examine the demands experienced by coworkers in German coworking spaces from a psychosocial and health-related point of view. Furthermore, coworking spaces were compared to the home office in order to answer the question whether coworking spaces are a better alternative to the home office. 

## 2. Theoretical Background

One possible approach of classifying the increasing flexibilization in the world of work has been proposed by Hofmann and Nøstdal [9]. According to the authors, flexibilization can be defined on three different levels: (1) The relocation of the organization’s risks, for example, by outsourcing or temporary work, which allows flexible cost reduction by increasing and decreasing the workforce at short notice; (2) the second level of flexibilization is represented by the amount and time of work in the context of task organization or working time models. This option offers different working models regarding the distribution of working hours (e.g., part-time or flex-time arrangements); (3) The third level described by the authors is the virtualization of work in terms of workplace location. The home office, mobile work, virtual work, virtual organizations, and the recently developed coworking spaces are located as virtualized working arrangements. 

According to this model, the present study focuses on two different workplace locations, namely home office and coworking space. While the first is a rather “traditional” approach to virtualized work, the latter can be seen as a new way of working where it becomes particularly evident how borders of traditional company offices dissipate. 

As the following explanations will show, working in a home office or coworking space is described by distinct features, except for both work arrangements being located beyond company offices.

### 2.1. Telework at the Home Office

According to the European social partners [10], telework is defined as ”a form of organizing and/or performing work, using information technology, in the context of an employment contract/relationship, where work, which could also be performed at the employers premises, is carried out away from those premises on a regular basis” (p. 2). 

Most of the national regulations on telework (e.g., the German Workplace Ordinance) include the main characteristics of this definition:

- Work is performed outside the company office on a regular basis;
- The use of information technology;
- The presence of an employer contract and, therefore;
- Exclusion of self-employed persons from being teleworkers, as they are not under an employment contract.

As stated by Brenke [11] 12% of German employees are working from home. Compared with other European countries Germany is in the mid-table, while for example more than 30% of the Swedish employees have the possibility for home-based telework [12]. Since not all kinds of work are suited for home-based execution (e.g., healthcare providers, production of goods), companies in the service branch are the main employers with a home office option. Regarding sociodemographic determinants in the prevalence of home-based telecommuting, there are no differences between men and women, little differences between households with children (14%) and without (11%) as well as moderate differences in work time (14% of full-time vs. 10% of part-time employees) [11]. Arnold & Steffens [13] consider 10% of all German employees as telecommuters who work from home during their working time. The dissemination of the possibility for home office is higher in bigger companies (>50 employees). The authors further describe that working in the home office is more common among jobs with higher qualification and responsibility. Moreover, white-collar workers (31%) work from home considerably more often on a regular basis than blue-collar workers (2%), probably resulting from the organization of the workplace which does not allow telework by nature.

### 2.2. Characteristics of Coworking Spaces

While the home office as a telework arrangement is mainly used by employed workers, coworking spaces are becoming an increasingly established work arrangement among self-employed and freelancers working in the creative line of business. In coworking spaces, an operator provides a workstation according to a certain fee schedule so that different people may share a common working environment. Coworking spaces usually consist of a wide open plan office that is accompanied by conference rooms, private offices, or cafés. The operator provides facilities such as access to the internet and in most cases office furniture such as desks and chairs [14,15,16]. Also, the operator may be responsible for community management which includes the organization of events or supporting programs. Coworking spaces vary significantly regarding equipment, philosophy, design, and rules depending on the preferences of coworking space operators as well as users. In this regard, several typologies of coworking spaces have been proposed. Spinuzzi [16] differentiates community workspaces, unoffices, and federated workspaces according to the underlying understanding of coworking, while Kojo & Nenonen [17] categorize coworking spaces based on the business model (profit/non-profit) and the level of access for users (public/semi-public/private). In the context of collaborative spaces of collaborative learning, coworking spaces are also considered to be economic-driven with a mix of user- and institution-led projects [18]. Generally, coworking can be seen as a third way between work in a conventional office and self-employed work at home or in public places [19]. Main characteristics of coworking spaces are collaboration, community, sustainability, openness, and accessibility [15]. These core values also reflect that coworking space can generally be seen as more than just sharing a physical space. Rather various forms of social participation and collaboration are encouraged and can be seen as defining elements [19], which may also facilitate innovative dynamics on several levels [20]. In this regard, positive developments concerning community building as well as urban development have been reported [21]. However, an increasing emergence of mixed forms between serviced offices and coworking spaces can be observed [22], which is also reflected in the increasing amount of private offices offered by coworking space providers [23].

According to the GSC, it is estimated that there are currently more than 19,000 coworking spaces operating worldwide providing working space for about 1.7 million people [24]. In Germany, approximately 300 spaces—mostly located in major cities—with 11,000 users can be assumed. A “typical” German coworking space has 68 members which is less than the worldwide average of 80 members. On average, there are 67 desks (70 desks is the worldwide average) [23,25].

With regard to the motives for working in a coworking space, creativity, networking, social interaction, and knowledge enrichment due to the collaboration with coworkers from different professional backgrounds are often named [26,27,28,29]. Participants of the GCS 2017 stated that the social and enjoyable atmosphere was the main reason for working in a coworking space, followed by interaction with others (56%) and community (55%). Seo et al. [30] also found relationship facilitation to be the most highly ranked attribute among coworking space users. Similarly, [16] Spinuzzi distinguishes good-neighbor and good-partner configurations of coworking spaces based on a more representative approach of loosely tied coworkers or a more inward facing core with ongoing networking and temporary collaboration. The coworking space as an alternative to the home office was the second most named reason for working in a coworking space indicated by coworkers in a qualitative study conducted by Servaty et al. [31]. Additionally, the flexibility offered by coworking spaces was appreciated by many self-employed because they do not need to bind themselves to rented office spaces via fixed contracts. In fact, coworking spaces rather allow flexible office use as well as access to important facilities, such as office equipment and internet access. In this regard, coworking spaces are an interesting solution for people who can independently create their own working hours and work environment [27,32].

Both, the home office and the coworking space are characterized by certain features concerning the psychosocial work environment which will be described within the next section in the light of the stress–strain concept.

### 2.3. The Concept of Stress and Strain

The concept of stress and strain originates in the research of Rohmert [33]; its center can be seen in a conceptual separation of working conditions and the work environment on the one hand, and an individual reaction to these factors on the other hand. Stressors are used as hypernym for all exogenous effects of the working system on employees. According to that, stress can be caused by characteristics of the work such as the task, equipment, and tools as well as physical or social factors. As a result and dependent on individual factors (constitution, abilities, and health status), (mental) strain develops. Thus, (mental) strain can be seen as the individual reaction to objective stressors. Experiencing strain for longer periods of time or with a certain intensity is assumed to be related to dysfunctions, diseases, or performance-related consequences. Stressors per se cannot and should not be avoided. Rather, there are promoting combinations of stressors that can lead to an optimal level of strain which is supposed to be associated with positive effects on health and performance [34,35].

The concept of stress and strain has found its way into international regulations in the form of DIN EN ISO 10075. Within this conceptualization, both stress and strain are understood in a neutral and value-free way. The interpretation of stress/stressors as stimuli that influence individuals must be separated linguistically and conceptually from psychophysiological stress reactions (strain). 

According to the stress–strain concept, the workplace, such as the home office or a coworking space, is associated with a certain combination of stressors which result in an individual strain reaction as the following examples show.

There was a positive effect between telework and autonomy as well as reconciliation of work and private life [36,37]. Mann and Holdsworth [38] determined that the feeling of being stressed was less among home-based teleworkers compared to traditional office workers. According to Allen et al. [39], the amount of home-based telework was negatively associated with work-related exhaustion. Additionally, having the option to work from home made employees feel less stressed, because of reduced commuting time and increased flexibility regarding child care [37].

With regard to work- and life-satisfaction, Morganson et al. as well as Brenke [4,11] found the highest rating among employees who worked from home on a regular basis, in contrast to other work arrangements. Similarly, a comparative study investigating employees in three different office settings (home office, virtual office, and traditional office) showed that the home office was the preferred model with regard to work-related aspects such as job performance as well as work–life balance [40].

Aside from positive effects of working in the home office where several factors such as autonomy and reconciliation seem to function as resources, there are also studies pointing out several difficulties related to the combination of stressors associated with working from home. That said, (social) isolation is a stressor often reported to be accompanying home-based work [38,39,41,42]. There is a significant increase in psychological stress and a reduction of work satisfaction with increasing isolation (hours of telework) as Bentley et al. [43] reported for a sample of home-based teleworkers in New Zealand. Additionally, professional isolation was also found to be related to lower performance; an effect that was also moderated by the time spent teleworking [41]. Besides, a considerable number of studies approved that social interaction at work can have a positive effect on strain and well-being [44,45,46]. 

Furthermore, work–life balance of teleworkers is described by a high amount of ambiguity [11]. Alongside the aforementioned positive effects, compatibility of work and private life may also be impaired for employees working from home, because of an aggravated separation of both domains and a blurring of boundaries (ibid). On the one hand, this includes the presence of family members and being accessible for them which may result in conflicts. Hence, the recreation phases as well as the family situation can be negatively affected by home-based telework [5,42]. On the other hand, “work” is constantly present at home which may increase working hours [11,12] and workload. Similarly, albeit reporting high satisfaction among teleworkers, Kelliher and Anderson [47] also found work intensification as a result of an exchange process in which employees raise their efforts for the flexibility granted by the employer. As a report of the European Union states, the amount of employees who feel usually or always stressed at work is higher among teleworkers than among those in the office at the organization [12]. All in all, these results highlight that flexible work arrangements, such as the home office, require high discipline and self-organization skills [48]. 

It becomes obvious that home-based work can be associated to positive strain reactions and can have positive effects on one’s health. Concurrently, the same factors which are advantageous in the first place can utter negative effects and bring up new challenges in this work environment. Therefore, it seems that quantity and frequency of telework using ICT should be following a defined set of rules in order to enhance positive outcomes [12]. 

No such results are currently available on the stressors experienced in coworking spaces and their health-related effects on its members [49]. Existing literature mostly has been taking on a descriptive focus. Nevertheless, these results have been pointing out to important topics that should be considered: The GCS 2017 illustrated that a quarter of all coworkers indicate noise as a problem in coworking spaces. Lack of privacy (19%), difficulties to concentrate (15%), and no social interaction (20%) were further issues named by the coworkers worldwide [8]. 

Furthermore, existing findings on positive and negative effects of working in the home office might be applicable to coworking spaces. Higher work–life balance and autonomy could also be beneficial in coworking spaces. Social isolation—named as a negative condition experienced when working from home—should not be as relevant for working in a coworking space or might even be considered beneficial in terms of social interaction. Self-organization could be easier in a coworking space due to structures provided compared to the home office. Although the setting created by the coworking space might also facilitate self-endangerment, because coworkers disregard their health and recreation phases [48].

It can be assumed that these aspects of the work environment “coworking space” are also affecting coworkers’ health and satisfaction. In order to prove these assumptions, empirical research is needed.

### 2.4. Objectives

The first part of the study investigated psychosocial working conditions and focused on stressors perceived by coworkers. Furthermore, overall satisfaction with the coworking space and subjective health status of coworkers were examined. Possible associations between perceived stressors and subjective satisfaction and health ratings were analyzed. 

Based on the assumptions made in the previous sections, the following hypotheses were proposed:

H1: In the coworking space, job stressors are negatively associated with (a) subjective health status of coworkers, (b) psychosomatic complaints, and (c) their satisfaction with working in a coworking space.

The second part of the analysis took into account the experiences of coworkers who previously worked (or partly work) in a home office setting and tried to answer the question whether coworkers evaluate coworking spaces better than the home office with regard to psychosocial and environmental working conditions, as well as health behavior and overall job satisfaction. Therefore, the following hypotheses were suggested:

H2: In comparison to the home office, the concept of coworking space attains higher levels of consent in respect to (a) perceived individual workability and performance (concentration, productivity, self-organization,), (b) psychosocial working conditions (social interaction, separation of work and private life, reconciliation of work and private life), (c) ergonomics and (d) health behavior (physical activity and eating behavior, breaks), and (e) job satisfaction.

H3: Compared to the home office, the coworking space model receives lower levels of consent with regard to (a) organizational working conditions (interruptions) and (b) environmental working conditions (privacy and noise).

## 3. Materials and Methods 

### 3.1. Study Design and Procedure

A quantitative study design with a cross-sectional approach via questionnaire was used for the present study. Data collection was based on a web-based survey that was conducted between June and October 2017. The survey software allowed for secure and anonymous data collection. 

The recruitment of coworkers was effected by means of coworking space providers. Therefore, an online search was carried out leading to the identification of 262 coworking space providers all across Germany. Among the coworking spaces contacted, there were “single” spaces but also multisite coworking spaces with several branches. In the following, providers were contacted via e-mail in which the purpose of the study was illustrated. Providers were asked to forward the study information as well as the weblink to the online-survey to all their members. Initially response rates were quite low so that telephone and e-mail reminders were implemented. Additionally, the study was promoted on social media platforms and the weblink to the study was posted on several internet portals. 

### 3.2. Variables and Instruments

The instrument included items that were based on a) valid instruments and b) newly developed, exploratory questions. The topics covered by the questionnaire included *sociodemographics*, *characteristics of the coworking space*, office environment, work organization, *stressors*, work engagement, *comparison of coworking space and home office*, *general health status, psychosomatic complaints*, and *satisfaction with the coworking space*. The focus of the present paper encompasses topics in italics. After a pretest, the questionnaire consisted of seven main parts with 54 items in total. The language of the questionnaire was German.

#### 3.2.1. Sociodemographics and Characteristics of the Coworking Spaces

In the first part, sociodemographic questions (gender, age, relationship, children) as well as questions on occupational qualification, employment type, and job sector referring to the first GCS (2010) were included [50]. Also, questions concerning the location of the coworking space were posed. The second part covered characteristics of the currently used coworking space, e.g., number of desks (*How many working places does the coworking space offer?*), desk type (*Are you using a “fixed-desk“ or a “flex desk”?*), coworking space layout (*In which room of the coworking space are you working most of the day (approx. 80%)?*), cooperation (*How often do you cooperate with other coworkers?*), duration (*How long have you been working in a coworking space?*), experience (*Have you been working in different coworking spaces previously?*), previously used work environments *(In which work environment have you been working prior to the coworking space?*). 

#### 3.2.2. Job Stressors

Job stressors as perceived by study participants were measured using several dimensions of the short questionnaire for work analysis (Kurzfragebogen zur Arbeitsanalyse, KFZA), namely qualitative workload (QLW), quantitative workload (QNW), work interruptions (WI), and work environment (WE) [51]. Each dimension was covered by two items that are summarized in scales by calculating a mean index. A 5-point Likert scale ranging from (1) *does not apply at all*, (2) *applies very little*, (3) *applies moderately*, (4) *mainly applies*, to (5) *totally applies* was used. 

Table 1 includes the corresponding items as well as coefficients for internal consistency measured by Spearman’s correlation in line with the original instrument. In comparison with the original instrument, coefficients obtained in the present study showed similar results.

Another question concerned possible negative consequences coworkers might experience due to demands. Therefore, five negative impacts were named allowing for multiple answers of participants (reduced productivity, reduced motivation, difficulties to concentrate, reduced working quality, other). There was also the possibility to indicate that no negative consequences resulted from the experienced demands.

#### 3.2.3. Health Status and Satisfaction with the Coworking Space Concept

The perceived health status was measured using a single item from the German version of the Copenhagen Psychosocial Questionnaire (COPSOQ,) rating from (0) *very bad* to (10) *very good* [52]. 

Furthermore, a scale on psychosomatic complaints for the nonclinical context [53] was used. A list of 20 psychosomatic complaints had to be rated with regard to their incidence ranging from (1) *never* to (5) *almost daily*. Statements on psychosomatic complaints were, for example, “*Do you have a headache?*” or “*Are you feeling tired and exhausted during the day?*” Internal consistency measured by Cronbach’s Alpha was found to be 0.70–0.93 in several studies, thus proving to be satisfactory to very good. In the current study with a very good α = 0.92 was attained.

Similar to the perceived health status, one item on satisfaction with working in the coworking space was applied ((0) *very unsatisfied* to (10) *very satisfied*). 

#### 3.2.4. Comparison of Coworking Spaces and Home Office

The comparison of the coworking space and the home office was based on aspects that were found to be potentially advantageous for either the coworking space (e.g., social support, separation of work and private life) or the home office (e.g., noise level, interruptions) according to the literature. Fourteen statements were phrased as can be seen in Table 2. Participants were asked to answer the statements using a 5-point Likert scale ranging from (1) *disagree* to (5) *agree*. Due to technical constraints within the survey software, a direct comparison of both work arrangements was not possible. Therefore, the coworking space—as the current workplace—was used as an anchor. 

### 3.3. Data Analysis

All computations for quantitative data analysis were performed using IBM Statistics SPSS 25. 

The first part of data analysis consisted of descriptive measures concerning sample characteristics and features of the currently used coworking space. Frequency distributions and measures of central tendency on perceived job stressors as well as health status, psychosomatic complaints, and satisfaction with the coworking space were analyzed. If possible, items were summarized into scales. Negative items were reversed for better interpretability. The question on negative consequences experienced because of job demands was transformed into a new metric variable based on the sum of negative consequences indicated (min. 0, max. 5).

Both, sociodemographic variables (gender, employment status) as well as features of the coworking space (desk type, office type) were assumed to be differentiating factors. Thus, either Mann-Whitney U-Test or independent t-test were applied where indicated. Missing values were excluded from the analysis, so that the respective number of cases is noted for all analyses performed. 

In the second part of data analysis, associations between job stressors and health status, psychosomatic complaints, and satisfaction with the coworking space were investigated, thus testing the proposed hypotheses. After testing for standard distribution (Kolmolgorov-Smirnov-Test), either Pearson’s correlation or Spearman’s rank correlation was used in order to determine correlation coefficients. A significance level of *p* = 0.05 (2-tailed) was assumed for the analysis. 

The comparison between home office and coworking space relied on frequency distributions to determine which aspects are seen as favorable in the home office or coworking space. For better interpretation, the 5-point-Likert scale was transformed into a 3-point scale by combining the positive (*rather agree* and *agree*) and negative ratings (*rather disagree* and *disagree*). A statement was seen as in favor of either the coworking space or home office if at least 50% of the respondents agreed on a positive item. Similarly, agreement rates of ≥50% on negative items were considered as disadvantageous, yet, they were not valued advantageous if not.

## 4. Results

In the following section, first, sample characteristics and results of the descriptive analysis will be presented. Secondly, results concerning the proposed hypotheses will be shown.

### 4.1. Sample Characteristics

There were 112 coworkers participating in the present study. Over half, 61.6%, of the participants were male. Mean age within the sample was M = 38.09 years (SD = 9.55 years). The majority of the coworkers lived in a relationship (69.6%) and 38.7% of them had children. With regard to employment status, most of the coworkers were self-employed (69.6%). The prevalent qualification was a university degree (59.8%). The main branches indicated by the participants were information technology and consulting (both 17.9%).

Concerning the duration of working in a coworking space (*n* = 97), most of the coworkers within the present sample used the coworking space for more than three months to one year (33%). Almost as many coworkers had been working in a coworking space between one and two years (27.8%), as there were coworkers with more than two years of coworking-experience (28.9%). Concerning the time spent in the coworking space (*n* = 86), the majority of the coworkers worked five days per week at the coworking space (32.6%). The average daily working time (*n* = 87) was for most coworkers about five to eight hours (52.9%). With regard to the previous place of work (*n* = 96), almost half of the participants (55.2%) had been working from home prior to working in a coworking space. Further information on the sample can be seen in Table 3. 

**Figure 1:**
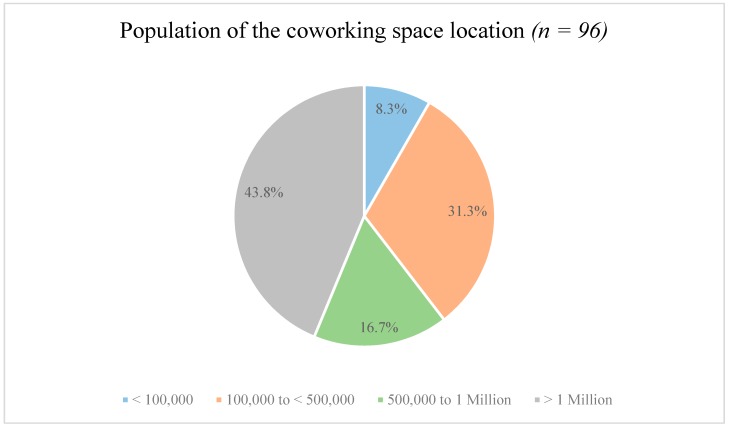
Population of the coworking space location.

Results on the location of the coworking space were available for 98 participants. With regard to the federal state, most of the coworkers were working in Berlin (18.4%), Hamburg (15.3%), or Bavaria (13.3). Federal states such as Mecklenburg-Hither Pomerania (1%) or Lower Saxony (1%) were less represented. As depicted in Figure 1, most coworkers said that the location of their coworking space had a population of >1 Million inhabitants. Only a small number of coworkers (8.3%) indicated to be working in cities with <100,000 inhabitants.

### 4.2. Descriptive Analysis

#### 4.2.1. Characteristics of the Coworking Space

In the present study a wide range of coworking spaces was presented as can be seen in Table 4. Data on the size of the coworking space was obtained by 96 participants: The most common coworking space size indicated was more than 40 desks (26.0%) as well as 10 to 20 workspaces (26.0%). Further information concerning the room type (*n* = 97) showed that the majority of the coworkers had been working in the open space (78.4%). Within the present sample, the fixed-desk model had been used more frequently (57.7%) than the flex-desk model (42.3%) which requires coworkers to look for a free desk before starting to work rather than to work at the same desk every day.

#### 4.2.2. Working Conditions—Job Stressors

Table 5 shows mean values and standard deviations for stressors experienced by coworkers. It can be seen that results were generally in a low range, except for quantitative workload which was located in a medium range.

Stressors were not found to be normally distributed, so that Mann-Whitney-U-Tests were used for comparison of means. According to the test, there were no statistical differences between desk-type (flex and fixed), office type (open and enclosed), and employment (self-employed and employed). Merely gender showed significant differences on the work environment scale (*p* = 0.048) with female coworkers perceiving more work interruptions (M = 2.00, SD = 0.88) than male coworkers (M = 1.64, SD = 0.79). Analysis of effect size resulted in a medium sized effect *η*^2^= 0.051 according to [54].

A total of 68 respondents answered the question concerning possible negative consequences resulting from experienced stressors. Analysis showed that 45.6% did not experience negative consequences resulting from experienced demands. Among the 54.4% of coworkers who indicated that there were negative consequences, difficulties to concentrate were named most often. Answering mode allowed multiple answers (max 5 negative consequences) but most participants only checked one (M = 1.38, SD = 0.59).

#### 4.2.3. General Health, Psychosomatic Complaints, and Satisfaction with the Coworking Space

With regard to their general health status, about half of the coworkers (50.7%) indicated a good health status (scores 7 to 8). A very good health status (scores 9 to 10) was reported by 29.5% of the coworkers in the present sample. Of the 71 respondents on this question, the mean value was M = 7.54 (SD = 1.2). There were no statistical differences between gender, employment type, office type, and desk-type.

Considering the psychosomatic complaints reported by coworkers, a low score was attained (M = 1.92, SD = 0.66) which showed that complaints are experienced on a very irregular basis (*every few months* as the verbal equivalent of a score of two). The question on fatigue (*Are you tiring quickly?*) obtained the highest rating with M = 3.01 (SD = 1.19). There were no statistical differences between gender, employment type, office type, and desk type.

When asked about their satisfaction with the coworking space, 61.1% of coworkers were very satisfied (scores 9 and 10) and another 34.3% were mostly satisfied (scores 7 and 8). This was also represented by M = 8.54 (SD = 1.35). There were no statistical differences between gender, office type and desk-type, and employment type. 

### 4.3. Associations between Working Conditions (Job Stressors), Subjective Health, Psychosomatic Complaints, and Satisfaction with the Coworking Space

The relationship between job stressors and subjective health status, psychosomatic complaints, as well as perceived satisfaction with the coworking space, was tested using Spearman’s rank correlation as a nonparametric test.

Hypothesis 1a assumed negative correlations between stressors and subjective health status. The analysis confirmed this relationship indicating that a higher workload (qualitative and quantitative) was associated with lower ratings of subjective health (r_QWL_ = −0.302, *p* < 0.05; r_QNW_ = −0.250, *p* < 0.05). Work interruptions and work environment were not significantly (*p* > 0.05) associated with the health status of the coworkers in this study. Hypothesis 1a was partly retained, because some of the stressors showed significant negative correlations with the subjective health status of coworkers as Table 6 shows. 

Furthermore, significant associations between job stressors and psychosomatic complaints were found. Especially qualitative workload and quantitative workload were positively correlated with psychosomatic complaints (r_QLW_ = 0.428, *p* = 0.000; r_QNW_ = 0.327, *p* < 0.01). Thus, the higher the workload (qualitative and quantitative) the more psychosomatic complaints were found. Hypothesis 1b was partly retained.

With regard to the association of job stressors and subjective satisfaction with the coworking space, a significant negative correlation was found. The more coworkers experienced an unfavorable working environment, the less satisfied they were with the coworking space (*r* = −0.310, *p* < 0.01). Therefore, hypothesis 1c was partially accepted. 

### 4.4. Comparison of Coworking Space and Home Office

**Figure 2:**
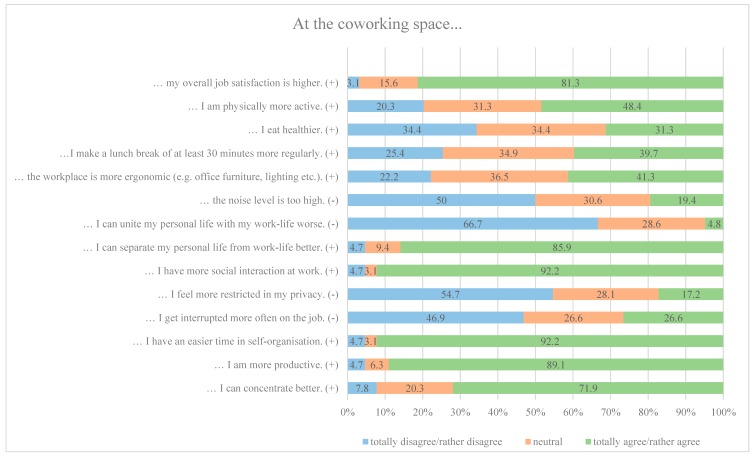
Comparison of working conditions at the coworking space and in the home office.

Hypothesis 2a, which assumed that perceived individual workability and performance in terms of concentration, productivity, and self-organization were more pronounced in the coworking space than in the home office, was accepted according to the 50% margin that was described in Section 3.3. As Figure 2 shows, 71.9% of the present sample rather agreed or totally agreed on the statement that they can better concentrate at the coworking space.

In line with hypothesis 2b, participants of the present study agreed to find more advantageous psychosocial working conditions in the coworking space. That is, social interaction (92.2%) and separation of work and private life (85.9%) were rated in favor of the coworking space. Concerning reconciliation of work and private life, which was negatively formulated, 66.7% of participants disagreed or rather disagreed to the statement indicating that the coworking space was the preferred workplace.

Figure 2 furthermore depicts that hypothesis 2c must be rejected, as the 50% margin of participants agreeing on the statement that the coworking space was more ergonomic in comparison to the home office was not reached (41.3%).

Hypothesis 2d which assumed a higher rating of health behavior in the coworking space must be rejected. Agreement ratings of eating behavior (31.3%), physical activity (48.4%), as well as breaks (39.7%) did not reach the 50% margin.

The majority of the study participants (81.3%) agreed to experience higher overall job satisfaction at the coworking space as compared to the home office. Thus, hypothesis 2e was accepted.

Hypothesis 3a focused on interruptions and assumed that participants agreed to being interrupted more often in the coworking space. Contrary to the hypothesis, only 26.6% of the sample described to be interrupted more often, so that hypothesis 3a had to be rejected.

Hypothesis 3b proposed that coworkers found the home office more favorable with regard to environmental working conditions, such as perceived privacy and noise. However, results showed that participants neither agreed to feel restricted in their privacy (17.2%) nor experienced too much noise (19.4%). Thus, hypothesis 3b must be rejected.

## 5. Discussion

The present exploratory study was able to show some interesting results. While generally encountering low to moderate perceived demands and being of good health, coworkers did feel less satisfied with working in the coworking space if the working environment was described as adverse. Furthermore, especially task-related stressors, such as qualitative and quantitative workload, were associated with subjective health as well as complaints. With regard to the comparison of home office and coworking space, the latter was found to be preferred in almost all inquired working conditions. In the following, some results are highlighted and discussed in-depth.

### 5.1. Working Conditions—Job Stressors

The present study did not find significant differences in the psychosocial demands experienced by employed and self-employed in the coworking space. With regard to their experiences concerning working from home, it can be assumed that self-employed homebased teleworkers suffered from blurring boundaries on the one hand and social isolation on the other hand, much as their employed counterparts [55], although the current definition of telework focuses on employees with an employment contract. 

Among the job stressors investigated within the study, quantitative workload was found to be most prominent, although still ranging in a moderate scope. This result is supported by other studies indicating that work and time pressures are quite common among the German workforce [56]. 

Qualitative workload obtained low scores. A possible explanation for these results might stem from the large part of self-employed and a generally high educational level. Both factors might contribute to the fact that the working tasks fit properly with abilities and competencies of job holders.

Furthermore, work interruption was found to be in the lower range. This is inconsistent with results from a representative study that reports work interruptions to be a major demand experienced by 44% of the respondents [56]. However, since the majority of the sample is self-employed, at least interruptions by colleagues should be occurring less often. Interruptions by other coworkers might be viewed as social exchange that is desired.

Coworkers stated that aspects of the work environment are experienced as minor demands. The respective questions comprised environmental conditions (noise, climate, dust) on the one hand and space equipment on the other hand. Rather positive evaluations of these conditions may be explained by the fact that coworkers generally decide voluntarily for the coworking space they want to use. Thus, they choose coworking spaces that appeal to them in terms of environmental conditions.

In sum, the demands imposed on coworkers are moderate to low. Thus, the coworking space generally offers good working conditions. However, as coworking spaces come in all shapes and sizes, it may be assumed that working conditions within the respective coworking spaces differ substantially. Furthermore, as the response rate within the present study was low, generalizability of results may be considered as restricted.

### 5.2. General Health, Psychosomatic Complaints, and Satisfaction with the Coworking Space

The present sample indicated, to be of good to very good health and psychosomatic complaints were found to be scarce. On the one hand this can be associated to the age of the participants which is comparatively young. In this regard, [56] showed that among persons in dependent employment only 14% describe their subjective health status as not so good or bad. Furthermore, this proportion increases with age. Similarly, a study investigating health-related quality of life within the German population found the ratings of general health to be decreasing with age [57]. 

On the other hand, employment status can be assumed as an influencing factor. In this regard, [58] found that healthier people are more likely to be engaged when self-employment. Within the course of self-employment, [59] found poor subjective health in a sample of freelance media workers which was associated to perceived effort–reward imbalance.

Since scientific studies within the context of coworking spaces are rare, there are no comparable results on the health status of coworkers. However, the present study relied on self-disclosure so that further research using more objective measures is encouraged. 

Satisfaction in the coworking space was found to be very high. These results are comparable to the results obtained by Servaty et al. [31]. In contrast, working in an open office space was found to be strongly related to a reduced job satisfaction [60], but coworking spaces seemed to be an exception. An aspect that might be attributed to the voluntariness and autonomy underlying most of the decisions to work in a coworking space as opposed to being assigned to a given workplace.

### 5.3. Associations between Job Stressors, General Health, Psychosomatic Complaints, and Satisfaction with the Coworking Space

In line with the stress–strain concept, several associations among perceived demands, such as qualitative workload and quantitative workload, and health, as well as psychosomatic complaints were found. Of 72 respondents who answered a question on the assumed influence of working in a coworking space on their health, 55.6% thought there was a positive influence. 

Among the job stressors, work environment was found to be the only dimension associated with satisfaction with the coworking space. While the scales on QNW and QLW are very task-related and thus capture only marginal aspects of the coworking space itself, demands imposed by WE are more attributable to the coworking space within the present context. Thus, it is noteworthy that WE were significantly associated with the satisfaction with the coworking space. The more adverse environmental factors are present, the lower the satisfaction with the coworking space. In terms of member turnover, this can be seen as an important aspect for coworking space operators. As the study by Seo et al. [30] showed, operators of coworking spaces attribute a high meaning to space and interior which was ranked second after relationship facilitation.

To sum up, based on the assumptions of the stress–strain concept, several associations between job stressors and health variables and satisfaction could be found. 

The results are in line with studies by Herbig et al. [61] and Kim and Dear [62] who found an effect of work stressors on the workers’ well-being due to the office layout (e.g., number of workers per enclosed room) but no positive effect or mediation of social interaction on workers’ well-being in association with office layout. However, at least when comparing the coworking space with the home office, the present sample did describe pronounced advantages with regard to social interaction as the following results show.

### 5.4. Comparison of Coworking Space and Home Office

The comparison of the coworking space with the home office environment indicates that many aspects are advantageous at the coworking space. Therefore, working at a coworking space could be a better alternative to the home office, but it is certainly not a worse one. The evaluation of the working situation at the coworking space by current coworkers in comparison with their experiences as (former or partly) home office users confirmed the positive characteristics of coworking spaces in many parts. 

Advantages of the coworking space were found to be very pronounced with regard to productivity, the ability to concentrate, and self-organization. This might seem contradictory in the light of possible distractions by other coworkers in the coworking space. But as the present study showed, when working in the home office, family members were often or always present during working hours in almost one third of the cases and only half of the participants reported to have a separated office/room to work at home so that the home office might also be an distracting working environment. Thus, it seems that the working conditions offered by the coworking space promote perceived performance as compared to the home office. This is in line with research from de Peuter et al. [63] where the productivity in a coworking space was highly appreciated, especially in comparison to working from home. However, it must be taken into account that the present data relied on self-report. In this regard and based on metrics of a performance management system, it has also been shown that transforming offices into open spaces was associated with reduced productivity [64]. 

All aspects referring to the social situation as well as compatibility of family and work were rated in favor of the coworking space which was expected. Social interaction remains one of the most important reasons for choosing to work in a coworking space. Although home office or telework are promising working models with regard to reconciliation of private and working life, studies found that working from home often is associated with a dissolution of boundaries and worse separation of work and private life [12]. However, some research also suggested that working in an open space office results in decreased face-to-face interaction and increased electronic interaction [64]. However, while projects for creating open space offices are often beyond employees’ decisions, the decision to work on a coworking space is based on voluntariness. The community offered by the coworking space and the associated social support can be considered as important resources, especially in comparison with the home office.

It is noticeable that within the present sample, 50% disagreed or rather disagreed with the statement *“in the coworking space the noise level is too high”*. If face-to-face communication decreases in open space offices [64], presumably the noise level decreases, too. This is in line with another result of the questionnaire according to which 53.9% of coworkers did not feel disturbed by undesirable speech sound. Similarly, although not meeting the 50% margin, 46.9% of the respondents answered that they were not interrupted more often in the coworking space than at home. This might be attributable to a decreasing face-to-face communication in open space offices, too. Additionally, the presence of family members during work at home might account for this result.

Interestingly, the majority of coworkers did not feel more restricted in their privacy as compared to the home office. This is a noticeable result since most of the coworkers said they worked in an open space within the coworking space which can be expected to be quite different from working at home. Futhermore, de Croon et al. [60] were able to show strong evidence for reduced psychological privacy in open workplaces. However, with regard to the motives for choosing to work in a coworking space, the social environment was named to be one of the main reasons. Thus, it might be argued that coworkers expect to work in an environment that is characterized by limited privacy and more prominent ambient noise. Nevertheless, as described by the GCS 2018, there is an increasing trend for coworking space providers to offer private offices [25] which means that coworkers may be looking for more enclosed and private working spaces. It can be assumed that privacy is preferred for working activities, which—at the same time—does not exclude the desire for the social environment offered by the coworking space. Furthermore, as was stated beforehand, working in a coworking space is based on a conscious choice and reduced privacy is to be expected. 

Participants of the present study described a better overall job satisfaction as compared to working in a home office. With regard to the results of Brenke [11] and Morganson et al. [4], indicating that people who regularly work from home show the highest work-and life-satisfaction scores among employees, this can be seen as noticeable. 

All in all, the comparison of coworking space and home office showed that coworkers prefer the working environment in coworking spaces especially regarding concentration, productivity, self-organization, social interaction, and separation of private and work life, as well as overall satisfaction.

### 5.5. Strengths and Limitations

There are several limitations related to the chosen study design. First, using a cross-sectional design does not allow for causal relations. In this regard, a longitudinal approach might have offered deeper insights into the impact of working conditions on health and satisfaction of coworkers. 

In the questionnaire, some scales and items showed only low reliability; partly, these ratings were found to be similar to the original studies. Nevertheless, general validity might be affected. 

The comparison of conditions experienced in the coworking space and the home office was only possible in relative terms because of technical constraints related to the survey software. As a consequence, statistical analysis was limited. Furthermore, ratings might be affected by recall bias since not all coworkers of the sample were using the coworking space and the home office parallel but had to rely on memory.

The present sample might succumb a selection bias via coworking space operators because they were responsible for the distribution of the survey within their coworking spaces. Coworking space provider could have influenced the composition of the sample twofold: Firstly, by not forwarding the study information at all and, secondly, by sending the study information only to the most engaged coworkers. Additionally, only coworkers who stayed within the coworking setting could be reached so that a survivor effect might be present. (Former) coworkers who decided against this type of work arrangement are not represented in the current sample. The remaining coworkers might have underlain self-serving bias in the completion of the questionnaire, because they voluntarily chose to work in the respective coworking space and had costs to pay which may influence their perceptions of the coworking space in general and comparisons with the home office experience in particular. 

Furthermore, the discussion of study results has to rely on a limited base of literature, because up to now there is only a small amount of scientific studies on coworking spaces. As the discussion showed, results on open space offices can be adduced for reference, although it must be taken into consideration that there are several preconditions that differ from working in a coworking space.

However, the present study was able to provide insights into a broad range of perceived working conditions of coworkers as well as their subjective health status. The latter has rarely been a subject of research up to date and thus provides meaningful insights into a relatively new mobile workforce. Results show that both perceived working conditions as well as subjective health status seem to be rather favorable. With regard to the increasing number of coworkers, this can be seen as an important result. However, since results are based on subjective assessments and perceptions, completion of the data by objective means (e.g., noise measurement, medical health checks) might further contribute to understanding this emerging working environment. The stress–strain concept which relies on a value-free understanding of stressors could be used for the description of working conditions. In terms of dependent variables, subjective health, complaints, and satisfaction with the coworking space proved to be beneficial variables, even if measured in a rather abridged form. Furthermore, the study was able to show that many coworkers used to work in the home office or are still doing so. Thus, comparing both working environments is a matter of practical and scientific relevance and the present results can be used as guidance for (future) coworkers and current teleworkers. As such, the study can be seen as a starting point for further research.

## 6. Conclusion

The present study examined coworking spaces from a psychosocial and health-related perspective and thus raises awareness for psychosocial working conditions in an emerging work environment. All in all, coworking spaces were found to be social and highly valued workplaces. In comparison to the home office, coworking spaces are perceived as the better workplace with regard to the majority of prompted items, but especially in terms of social aspects, self-organization and perceived productivity as well as overall satisfaction. Even noise and privacy issues were not found to be explicitly worse than in the home office. Only for work interruptions and aspects related to health behavior there were not any clear preferences. In this regard, an informed decision to work in a coworking space must take into account that factors such as noise, interruptions, and reduced privacy can be present. For operators of coworking spaces, these aspects, as well as ergonomics, offer potential for improvement in their effort for providing healthy workplaces. The trend of an increasing amount of private offices observed by the GCS may account for coworkers’ wishes for less noise and more privacy [25]. In addition to such a spatial approach, further possibilities to account for noise and privacy include rules or schedules for quieter/more silent working hours or spatial separation of common and work spaces.

Research on open space offices can be used to offer some explanations of the present results. In this regard, reduced face-to-face communication and an increase in electronic communication that were observed with the dissolution of physical barriers in an office [64] might be a reason why noise did not prove to be a prominent stressor for the sample of coworkers. Concurrently, research on open space offices does not cope entirely with working conditions in a coworking space as the results on satisfaction show. Although voluntariness was not investigated in the study, it can be assumed to be a moderating factor between demands imposed by the working environment and perceived health and satisfaction. Thus, while some characteristics of open space offices and coworking spaces seem to be comparable, the resulting working conditions and strain reactions diverge. Predictions based on research on open space offices must be treated with caution.

Further studies might take a more comprehensive view on working conditions focussing on further stressors as well as resources that are experienced by coworkers and their resulting health and satisfaction. A more detailed analysis of different user types is encouraged. In this regard, the psychosocial working conditions and strain reactions of employees of small and medium sized businesses should be examined. Does it make a difference to be working in a coworking space as a set team or complete business? In this regard [65], see the potential for changing group dynamics when introducing affiliated coworkers in a coworking space. However, coworking spaces seem to gain increasing attractiveness for employed persons, making some coworking spaces comparable to the early mentioned neighborhood work centers in which several companies share office spaces within a building. While this might reduce some negative effects of employed telework such as social isolation, other aspects such as a firm-specific learning environment and organizational culture might still be lacking [66]. Thus, for companies it is important to take into consideration the respective challenges and benefits. Additionally, legal issues with regard to workplace risk assessments must be taken into consideration.

This study gives first insights into the psychosocial environment of coworking spaces and their users’ health and satisfaction with the coworking space. Nevertheless, many questions remain unanswered so that justified recommendations for coworking space providers and political decision makers need further comprehensive research.

More research and health-promotion strategies especially tailored for this rather flexible work environment need to be implemented, so that coworking spaces can permanently establish themselves as a healthy, alternative work arrangement.

### Practical Implications

Coworking spaces as new emerging workplaces were found to be highly valued in the present sample, especially in comparison to the home office. Nevertheless, the present study showed several practical implications for coworkers and operators of coworking spaces that can enhance coworkers’ experience while offering a health-promoting working environment: Coworking space operators can offer workshops or trainings for skill development of which self-employed (within the same branch) could benefit.

Furthermore, space and adequate office furniture were not found to be a standard feature in the (former) home office of coworkers. Thus, coworking space operators should emphasize these aspects to attract users and offer an ergonomic, health promoting working environment. In this regard, some coworkers of the present study indicated that a professional workplace was one of the reasons for choosing to work in a coworking space. In this line, work environment was significantly associated with satisfaction and can thus be seen as an important topic for coworking space operators. They should engage in designing appealing rooms and facilities as well as keeping an eye on environmental factors such as noise in order to reduce turnover and improve their members’ satisfaction.

## Figures and Tables

**Table 1:** Items and measures of internal consistency for the demands.

Dimension	Items	Internal Consistency (*r*) by Prümper et al. (1995)	Internal Consistency (*r*) Current Study
Qualitative workload	There are things in my work, which are too complicated (e.g., because of no or unclear job specification or insufficient qualification).	0.40	0.45**
	There are too high demands on my ability to concentrate.		
Quantitative workload	I am often pressed for time.	0.70	0.71**
	I have too much work.		
Work interruptions	I am often lacking required information, materials, and work equipment.	0.44	0.36**
	I consistently get interrupted during my work by other persons.		
Work environment	There are adverse environmental factors at my work place like noise, climate, and dust.	0.60	0.58**
	Rooms and room facilities are insufficient at my work place.		

** the correlation is significant *p* = 0.01 (2-tailed).

**Table 2:** Items used for comparing the coworking space and the home office.

Items At the Coworking Space, …
… I can concentrate better. (+)
… I have an easier time in self-organization. (+)
… I get more often interrupted on the job. (-)
… I feel more restricted in my privacy. (-)
… I am more productive. (+)
… I have more social interaction at work. (+)
… I can separate my personal life from work-life better (+)
… I can unite my personal life with my work-life worse. (-)
… the noise level is too high. (-)
… the workplace is more ergonomic (e.g., office furniture, lighting). (+)
… I make a lunch break of at least 30 minutes more regularly. (+)
… I eat healthier. (+)
… I am physically more active. (+)
… my overall job satisfaction is higher. (+)

**Table 3:** Sample characteristics.

**Sociodemographics**		***n***	**%**
Gender (*n* = 112)	male	69	61.6
	female	43	38.4
Age (*n* = 111)	≤29	23	20.7
	30–39	56	41.5
	40–49	24	25.2
	≥50	14	12.6
Relationship status (*n* = 112)	In a relationship	78	69.6
	Not in a relationship	34	30.4
Children (*n* = 112)	Yes	43	38.7
	No	68	61.3
Employment status (*n* = 112)	Self-employed	78	69.6
	Employed	34	30.4
Qualification (*n* = 112)	None	4	3.6
	Apprenticeship (dual system)	6	5.4
	Professional school, technical school, or vocational academy	15	13.4
	University of applied sciences degree	13	11.6
	University degree	67	59.8
	Doctorate	3	2.7
	other	4	3.6
Main branches (*n* = 112)	IT	20	17.9
	Consulting	20	17.9
	Artwork (graphic- or webdesign, photography)	10	8.9
	Media/journalism	10	8.9
	Public relations/marketing	9	8.0
	other	37	33.1
**Characteristics of working in a coworking space**		***n***	**%**
Duration of working in a coworking space (*n* = 97)	<3 months	10	10.3
	3 months–<1 year	32	33.0
	1–2 years	27	27.8
	>2 years	28	28.9
Number of days per week working in the coworking space (*n* = 86)	1	9	10.5
	2	7	8.1
	3	17	19.8
	4	20	23.3
	5	28	32.6
	6	4	4.7
	7	1	1.2
Hours per day working in the coworking space (*n* = 87)	<5 h	2	2.3
	5–8 h	46	52.9
	9–10 h	31	35.6
	>10 h	8	9.2

**Table 4:** Characteristics of the coworking space.

Coworking Space Characteristics		*n*	%
Size of the coworking space in number of desks (*n* = 96)	<10	11	11.5
	10–20	25	26.0
	21–30	21	21.9
	31–40	14	14.6
	>40	25	26.0
Office type in the coworking space (*n* = 97)	Open space	76	78.4
	Team office (min. 2 persons)	19	19.6
	Single office	1	1.0
	Conference room	1	1.0
Desk type in the coworking space (*n* = 97)	Flex desk	41	42.3
	Fix desk	56	57.7

**Table 5:** Means and standard deviations of demands.

Demand	Mean/Standard Deviation
Qualitative workload	M = 1.98, SD = 0.80 (*n* = 73)
Quantitative workload	M = 2.86, SD = 0.96 (*n* = 73)
Work interruptions	M = 2.13, SD = 0.77 (*n* = 73)
Work environment	M = 1.77, SD = 0.83 (*n* = 72)

**Table 6:** Correlations between demands and subjective health status, psychosomatic complaints, and satisfaction with the coworking space.

Demand	Subjective Health Status	Psychosomatic Complaints	Satisfaction with the Coworking Space
Qualitative Workload	*r* = −0.302 *, *p* = 0.011 (*n* = 70)	*r* = 0.428 **, *p* = 0.000 (*n* = 70)	*r* = −0.232, *p* = 0.053 (*n* = 70)
Quantitative workload	*r* = −0.250 *, *p* = 0.036 (*n* = 70)	*r* = 0.327 **, *p* = 0.006 (*n* = 70)	*r* = −0.208, *p* = 0.085 (*n* = 70)
Work interruptions	*r* = −0.167, *p* = 0.166 (*n* = 70)	*r* = 0.173, *p* = 0.152(*n* = 70)	*r* = −0.233, *p* = 0.052 (*n* = 70)
Work environment	*r* = −0.145, *p* = 0.232 (*n* = 70)	*r* = 0.050, *p* = 0.684 (*n* = 70)	*r* = −0.310 **, *p* = 0.009 (*n* = 70)

* *p* < 0.05, 2-tailed; ** *p* < 0.01, 2-tailed.

## Note

The paper is based on the results of HKs unpublished Master Thesis carried out at the Hamburg University of Applied Sciences.

## References

[1] Mache S., Harth V. (2016). Flexibilisierte Arbeitsformen. Zentralblatt für Arbeitsmedizin Arbeitsschutz und Ergonomie.

[2] Bailey D.E., Kurland N.B. (2002). A review of telework research: Findings, new directions, and lessons for the study of modern work. J. Organ. Behav. Int. J. Ind. Occup. Organ. Psychol. Behav..

[3] Messenger J., Gschwind L. (2016). Three Generations of Telework: New ICTs and (R)evolution from Home Office to Virtual Office. New Technol. Work Employ..

[4] Morganson V.J., Major D.A., Oborn K.L., Verive J.M., Heelan M.P. (2010). Comparing telework locations and traditional work arrangements: Differences in work-life balance support, job satisfaction, and inclusion. J. Manag. Psychol..

[5] Rasmussen E., Corbett G. (2008). Why isn’t teleworking working?. N. Z. J. Employ. Relat..

[6] Valenduc G., Vendramin P. (2001). Telework: From distance working to new forms of flexible work organisation. Transf. Eur. Rev. Labour Res..

[7] Keller H., Robelski S., Harth V., Mache S. (2017). Psychosoziale Aspekte bei der Arbeit im Homeoffice und in Coworking Spaces. ASU Arb. Soz. Umweltmed..

[8] Deskmag The 2017 Global Coworking Survey. Ultimate Member Data: Utilization of Coworking Spaces. www.slideshare.net/carstenfoertsch/utilization-of-coworking-spaces-members-of-coworking-spaces-part-2-of-2-80912960.

[9] Hofmann J., Nøstdal R. (2014). Einsatz und Bedeutung Externen Spezialisten.

[10] ETUC, UNICE, UEAPME, CEEP (2002). Framework Agreement on Telework: Report by the European Social Partners.

[11] Brenke K. (2016). Home Office: Möglichkeiten werden bei weitem nicht ausgeschöpft: DIW Wochenbericht. Ger. Inst. Econ. Res..

[12] Eurofound and the International Labour Office (2017). Working Anytime, Anywhere: The Effects on the World of Work.

[13] Arnold D., Steffens S. (2016). Arbeiten zu Hause: Verbreitung, Ausgestaltung und Bewertung. Eine repräsentative Bestandsaufnahme für die deutsche Privatwirtschaft mittels verknüpfter Arbeitgeber-Arbeitnehmer Daten. Betriebliche Prävention.

[14] Pohler N. (2012). Neue Arbeitsräume für neue Arbeitsformen: Coworking Spaces. Österreichische Zeitschrift für Soziologie.

[15] Schürmann M. (2013). Coworking Space: Geschäftsmodell für Entrepreneure und Wissensarbeiter.

[16] Spinuzzi C. (2012). Working Alone Together. J. Bus. Tech. Commun..

[17] Kojo I., Nenonen S. (2016). Typologies for co-working spaces in Finland–what and how?. Facilities.

[18] Capdevila I. Typologies of Localized Spaces of Collaborative Innovation. https://ssrn.com/abstract=2414402.

[19] Waters-Lynch J., Potts J., Butcher T., Dodson J., Hurley J. Coworking: A Transdisciplinary Overview. https://ssrn.com/abstract=2712217.

[20] Capdevila I. (2015). Co-working spaces and the localised dynamics of innovation in Barcelona. Int. J. Innov. Manag..

[21] Akhavan M., Mariotti I., Astolfi L., Canevari A. (2019). Coworking Spaces and New Social Relations: A Focus an the Social Streets in Italy. Urban Sci..

[22] Mariotti I., Pacchi C., Di Vita S. (2017). Co-working Spaces in Milan: Location Patterns and Urban Effects. J. Urban Technol..

[23] Deskmag Coworking in Deutschland. http://www.deskmag.com/de/coworking-spaces-in-deutschland-2018-marktreport-studie-erhebung-993.

[24] Foertsch C. 1.7 Millionen Mitglieder Werden 2018 Weltweit in Coworking Spaces Arbeiten. http://www.deskmag.com/de/1-7-millionen-mitglieder-werden-2018-in-coworking-spaces-arbeiten-weltweite-umfrage-studie-marktberi.

[25] Foertsch C. The 2018 State of Coworking Spaces. http://www.deskmag.com/en/the-state-of-coworking-spaces-in-2018-market-research-development-survey.

[26] Döring S. (2010). Zusammen Flexibel ist Man Weniger Allein? Eine Empirische Analyse der Neuen Arbeitsform Coworking als Möglichkeit der Wissensgenerierung.

[27] Gandini A. (2015). The rise of coworking spaces: A literature review. Ephemer. Theory Politics Organ..

[28] Garrett L.E., Spreitzer G.M., Bacevice P.A. (2017). Co-constructing a sense of community at work: The emergence of community in coworking spaces. Organ. Stud..

[29] Gerdenitsch C., Scheel T.E., Andorfer J., Korunka C. (2016). Coworking spaces: A source of social support for independent professionals. Front. Psychol..

[30] Seo J., Lysiankova L., Ock Y.-S., Chun D. (2017). Priorities of coworking space operation based on comparison of the hosts and users’ perspectives. Sustainability.

[31] Servaty R., Perger G., Harth V., Mache S. (2018). Working in a cocoon: (Co) working conditions of office nomads–a health related qualitative study of shared working environments. Work.

[32] Bouncken R.B., Reuschl A.J. (2018). Coworking-spaces: How a phenomenon of the sharing economy builds a novel trend for the workplace and for entrepreneurship. Rev. Manag. Sci..

[33] Rohmert W. (1984). Das Belastungs-Beanspruchungs-Konzept. Zeitschrift für Arbeitswissenschaft.

[34] Joiko K., Schmauder M., Wolff G. (2010). Psychische Belastung und Beanspruchung im Berufsleben: Erkennen—Gestalten.

[35] Rosen P.H., Wischniewski S. (2019). Scoping review on job control and occupational health in the manufacturing context. Int. J. Adv. Manuf. Technol..

[36] Harpaz I. (2002). Advantages and disadvantages of telecommuting for the individual, organization and society. Work Study.

[37] Gajendran R.S., Harrison D.A. (2007). The good, the bad, and the unknown about telecommuting: Meta-analysis of psychological mediators and individual consequences. J. Appl. Psychol..

[38] Mann S., Holdsworth L. (2003). The psychological impact of teleworking: Stress, emotions and health. New Technol. Work Employ..

[39] Allen T.D., Golden T.D., Shockley K.M. (2015). How effective is telecommuting? Assessing the status of our scientific findings. Psychol. Sci. Public Interest.

[40] Hill J., Ferris M., Märtinson V. (2003). Does it matter where you work? A comparison of how three work venues (traditional office, virtual office, and home office) influence aspects of work and personal/family life. J. Vocat. Behav..

[41] Golden T.D., Veiga J.F., Dino R.N. (2008). The impact of professional isolation on teleworker job performance and turnover intentions: Does time spent teleworking, interacting face-to-face, or having access to communication-enhancing technology matter?. J. Appl. Psychol..

[42] Harris L. (2003). Home-based teleworking and the employment relationship: Managerial challenges and dilemmas. Pers. Rev..

[43] Bentley T.A., Teo S.T.T., McLeod L., Tan F., Bosua R., Gloet M. (2016). The role of organisational support in teleworker wellbeing: A socio-technical systems approach. Appl. Ergon..

[44] Danna K., Griffin R.W. (1999). Health and well-being in the workplace: A review and synthesis of the literature. J. Manag..

[45] Demerouti E., Bakker A.B. (2011). The Job Demands–Resources model: Challenges for future research. SA J. Ind. Psychol..

[46] Drössler S., Steputat A., Schubert M., Euler U., Seidler A. (2016). Psychische Gesundheit in der Arbeitswelt. Soziale Beziehungen.

[47] Kelliher C., Anderson D. (2010). Doing more with less? Flexible working practices and the intensification of work. Hum. Relat..

[48] Krause A., Baeriswyl S., Berset M., Deci N., Dettmers J., Dorsemagen C., Meier W., Schraner S., Stetter B., Straub L. (2014). Selbstgefährdung als Indikator für Mängel bei der Gestaltung mobil-flexibler Arbeit: Zur Entwicklung eines Erhebungsinstruments. Wirtschaftspsychologie.

[49] Servaty R., Harth V., Mache S. (2016). Arbeitsbedingungen in Coworking Spaces unter motivationalen und gesundheitsrelevanten Aspekten. Zentralblatt für Arbeitsmedizin Arbeitsschutz und Ergonomie.

[50] Foertsch C. The Coworker’s Profile. www.deskmag.com/en/the-coworkers-global-coworking-survey-168.

[51] Prümper J., Hartmannsgruber K., Frese M. (1995). KFZA. Kurzfragenbogen zur Arbeitsanalyse. Zeitschrift für Arbeits und Organisationspsychologie.

[52] Nübling M., Stößel U., Hasselhorn H.-M., Hofmann F. (2005). Methoden zur Erfassung Psychischer Belastungen. Erprobung Eines Messinstrumentes (COPSOQ).

[53] Mohr G., Müller A. (2014). Psychosomatische Beschwerden im nicht-klinischen Kontext. Zusammenstellung Sozialwissenschaftlicher Items und Skalen.

[54] Cohen J. (1988). Statistical Power Analysis for the Behavioral Sciences.

[55] Tremblay D.-G., Genin É. (2007). The demand for telework of IT self-employed workers. J. E-Work..

[56] Lohmann-Haislah A. (2012). Stressreport Deutschland 2012: Psychische Anforderungen, Ressourcen und Befinden.

[57] Ellert U., Kurth B.M. (2013). Gesundheitsbezogene Lebensqualität bei Erwachsenen in Deutschland. Bundesgesundheitsblatt Gesundheitsforschung Gesundheitsschutz.

[58] Rietveld C.A., van Kippersluis H., Thurik A.R. (2015). Self-employment and health: Barriers or benefits?. Health Econ..

[59] Ertell M., Proell U. (2008). Selbstständig und gesund in freiberuflicher Tätigkeit. Freie Berufe—Gestalter der Gesellschaft. Festschrift zum 60-jährigen Bestehen des Verbandes Freier Berufe im Lande NRW e.V..

[60] de Croon E.M., Sluiter J.K., Kuijer P.P.F.M., Frings-Dresen M.H.W. (2005). The effect of office concepts on worker health and performance: A systematic review of the literature. Ergonomics.

[61] Herbig B., Schneider A., Nowak D. (2015). Does office space occupation matter? The role of the number of persons per enclosed office space, psychosocial work characteristics, and environmental satisfaction in the physical and mental health of employees. Indoor Air.

[62] Kim J., Dear R. (2013). Workspace satisfaction: The privacy-communication trade-off in open-plan offices. J. Environ. Psychol..

[63] De Peuter G., Cohen N.S., Saraco F. (2017). The ambivalence of coworking: On the politics of an emerging work practice. Eur. J. Cult. Stud..

[64] Bernstein E.S., Turban S. (2018). The impact of the ‘open’workspace on human collaboration. Philos. Trans. R. Soc. B Biol. Sci..

[65] Ross P., Ressia S. (2015). Neither office nor home: Coworking as an emerging workplace choice. Employ. Relat. Rec..

[66] Kurland N.B., Bailey D.E. (1999). Telework: The advantages and challenges of working here, there, anywhere, and anytime. Organ. Dyn..

